# Anticoagulation Pauses and Anesthetic Options: A Case for Multidisciplinary Harmonization

**DOI:** 10.7759/cureus.107704

**Published:** 2026-04-25

**Authors:** Breethaa Janani Selvamani, Rakesh V Sondekoppam

**Affiliations:** 1 Anesthesiology and Perioperative Medicine, Mayo Clinic, Rochester, USA; 2 Anesthesiology, Stanford University, Palo Alto, USA

**Keywords:** anticoagulants, antithrombotics, direct oral anticoagulant (doac), doac, nerve blocks, regional anesthesia

## Abstract

Perioperative anticoagulation management is increasingly guided by recommendations from multiple specialties, including cardiology, thrombosis medicine, stroke medicine, and surgery. These frameworks, while intending to improve standardization of interruption and resumption strategies for antithrombotic therapy, are largely centered on surgical bleeding risk and thromboembolic risk, while regional anesthesia (RA) guidelines differ in pause intervals for many oral anticoagulants due to the differing thresholds for acceptable residual anticoagulant activity, with the potentially catastrophic consequences of neuraxial hematoma in mind. Emerging evidence also suggests that measurable direct oral anticoagulant (DOAC) activity may persist despite guideline-consistent interruption in some patients, highlighting pharmacokinetic variability in real-world perioperative populations. Multidisciplinary, institution-specific frameworks and testing of anticoagulant activity in select cases may help harmonize multiple sources of guidance and support coordinated decision-making across surgical, anesthesia, and perioperative care teams. In this perspective piece, we intend to highlight discrepancies in perioperative anticoagulation guidance and discuss ways to promote harmonization strategies with considerations for RA choices.

## Editorial

An abundance of guidance does not necessarily translate into clarity. When recommendations diverge, clinicians are left navigating between competing interpretations of acceptable risk. Perioperative anticoagulation management is increasingly shaped by guidance from multiple specialties such as cardiology, hematology, stroke medicine, and surgery [1-4]. These frameworks have improved structure around interruption and resumption strategies for anti-thrombotic therapy. However, they are largely constructed around two primary considerations: surgical bleeding risk and thromboembolic risk. For the regional anesthesiologist, an additional dimension must be incorporated, which is the safety and feasibility of the intended anesthetic technique. This editorial examines discrepancies across specialty guidelines and their implications for regional anesthesia (RA) and advocates for institution-specific harmonization of anticoagulation interruption strategies to preserve RA as a viable option.

Anesthesia-specific guidance, including that from the American Society of Regional Anesthesia and Pain Medicine (ASRA) and the European Society of Anaesthesia and Intensive Care/European Society of Regional Anaesthesia and Pain Therapy (ESAIC/ESRA), is anchored in preventing neuraxial and deep plexus hematoma, a rare but potentially devastating complication [5, 6]. In contrast, cardiology-derived strategies such as the Perioperative Anticoagulant Use for Surgery Evaluation (PAUSE) protocol or those that are aimed at stroke prevention emphasize pharmacokinetic predictability and bleeding-risk stratification, supporting shorter interruption intervals for many procedures [1-4, 7]. As discussed recently [7], this discrepancy, where ASRA recommends a minimum 72-hour interruption before neuraxial procedures, while the PAUSE trial-derived strategies to hold direct oral anticoagulants (DOACs) approximately for 60-68 hours for high-bleeding-risk surgery, while internally coherent, reflects divergence in what is considered acceptable residual risk. Although surgical site hematomas can be clinically significant, they are generally detectable and amenable to timely management; in contrast, neuraxial hematomas may present insidiously and, even when recognized, carry a high risk of permanent neurologic sequelae. This distinction underpins the greater conservatism of anesthesia-focused recommendations. A wound hematoma and an epidural hematoma differ in consequences.

Differences in anticoagulation interruption strategies across specialty guidelines may create uncertainty for clinicians and patients alike (Table 1).

**Table 1:** Commonalities and discrepancies in the perioperative anticoagulation guidance across major guidelines PAUSE: Perioperative Anticoagulant Use for Surgery Evaluation trial; ACCP: American College of Chest Physicians; ASRA-PM: American Society of Regional Anesthesia and Pain Medicine; ESAIC: European Society of Anaesthesia and Intensive Care; ESRA: European Society of Regional Anesthesia; h: Hours; DOAC: Direct Oral Anticoagulants; CrCl: Creatinine Clearance; VKA: Vitamin-K Antagonists; INR: International Normalized Ratio; ASA: Acetylsalicylic Acid; LMWH: Low Molecular Weight Heparin; SC: Subcutaneous; aPTT: Activated Partial Thromboplastin Time

Drug/Class	PAUSE Study [1]	Thrombosis Canada [2]	Canadian Stroke Best Practice [3]	CHEST/ACCP 2022 [4]	ESAIC/ESRA 2022 [6]	ASRA-PM 2025 [5]
Apixaban/ Rivaroxaban/ Edoxaban (DOACs)	Low bleeding risk: hold 1 day before procedure. High bleeding risk: hold 2 days before. Resume 1 day after low-risk or 2–3 days after high-risk surgery.	Low–moderate bleeding risk: hold 2 days before. High bleeding risk or neuraxial: hold 3 days before. Resume 24 h after low-risk or 48–72 h after high-risk procedures.	Low–moderate bleeding risk: hold 1 day before. High bleeding risk: hold 2 days before. Resume ≥24 h surgery.	Stop 1–2 days before surgery, depending on bleeding risk; resume 24 h (low risk) or 48–72 h (high risk) postoperatively.	Low-dose DOAC: hold rivaroxaban/edoxaban 24 h, apixaban 36 h prior to neuraxial or deep nerve blocks. High-dose DOAC: hold ≥72 h before block. Restart only after catheter removal.	Low-dose DOAC: rivaroxaban/edoxaban 24 h, apixaban 36 h before neuraxial/deep nerve blocks. High-dose DOAC: ≥72 h before block. Restart 6 h if low dose or 24 h if high dose after catheter removal.
Dabigatran	Similar framework to other DOACs, but interruption is extended with impaired renal function.	Low-moderate bleeding risk: hold 2-3 days depending on renal function; High bleeding risk: hold 3–5 days before surgery, depending on renal function. Resume 24 h after low-risk or 48–72 h after high bleeding risk procedures.	Standard DOAC interruption plus an additional 1–2 days if CrCl <50 mL/min (up to ~4 days before high-risk surgery).	Stop 1–4 days before surgery, depending on renal function. Resume 24–72 h after surgery, depending on bleeding risk.	Low-dose: hold 48 h before neuraxial or deep nerve blocks. High dose: hold 72 h	72 h before neuraxial block if CrCl ≥50; 120 h if CrCl 30–49. Restart 6 h if low dose or 24 h if high dose after catheter removal.
Warfarin (VKA)	Not specifically addressed.	Hold ~5 days before surgery and check INR to ensure 1.5 or less; resume postoperatively when the patient is drinking fluids, consider a bolus dose	Hold 5 days before surgery; resume within 24 h postoperatively in most patients. Selected high-stroke-risk patients may receive bridging therapy.	Generally hold ³5 days before surgery; bridging is reserved for selected high-thromboembolic-risk patients; Resume within 24 h after surgery	Hold 5 days and normal INR before neuraxial or deep nerve blocks. Indwelling catheters are contraindicated.	Hold for 5 days and normal INR before neuraxial /deep nerve blocks; Indwelling catheters may be maintained or removed with caution with low-dose warfarin therapy. Monitor INR and remove the catheter when INR < 1.5. Resume immediately following catheter removal.
Aspirin (ASA)	Not addressed.	Continue for most procedures. Consider holding 7 days before very high-bleeding-risk surgery (e.g., intracranial).	Continue for most procedures. High-bleed-risk procedures: interrupt 7–10 days before; Continue in patients with high cardiovascular risk	Suggest continuing aspirin for many elective noncardiac surgeries rather than interrupting therapy.	Low-dose aspirin-no interruption. High-dose aspirin- hold 3 to 7 days before neuraxial or deep nerve blocks.	No mandatory pause for aspirin alone for neuraxial/deep nerve block procedures.
P2Y12 inhibitors (Clopidogrel / Ticagrelor / Prasugrel)	Not addressed.	Hold clopidogrel 5 days, ticagrelor 3-5 days, and prasugrel 7 days before surgery.	Not addressed	Typical interruption: clopidogrel 5 days, ticagrelor 3–5 days, prasugrel 7 days before surgery. Resume 24 h after the procedure.	Hold clopidogrel 5–7 days, ticagrelor 5 days, and prasugrel 7 days before neuraxial or deep nerve blocks. Indwelling catheters are contraindicated.	Hold clopidogrel 5–7 days, ticagrelor ~5 days, prasugrel 7–10 days before neuraxial/deep nerve block. Indwelling catheters may be maintained for 1-2 days with clopidogrel, provided a loading dose is not administered. Resume immediately if no loading dose; Hold 6 hours after catheter removal if administering a loading dose.
LMWH – Low dose	Not addressed.	Interruption not needed routinely; Follow ASRA/ESRA guidelines if neuraxial anesthesia is planned.	Guidance focuses mainly on bridging therapy; low-dose LMWH may be used postoperatively for 1–3 days in selected patients who cannot take oral therapy.	Guidance focuses mainly on postoperative bridging therapy for the initial 2-3 days in high bleeding risk procedures.	Hold ≥12 h before neuraxial or deep nerve blocks.	Hold ≥12 h before neuraxial/deep nerve blocks; restart ≥4 h after catheter removal.
LMWH – High dose	Not addressed.	Once daily dose-hold 2 days; Twice daily dose-hold 1 day; Follow ASRA/ESRA if neuraxial anesthesia planned	LMWH is mainly discussed as bridging therapy in selected high-thromboembolic-risk patients receiving warfarin; postoperative bridging may be omitted after high-bleeding-risk procedures.	Guidance focuses mainly on bridging therapy; Hold 24 h before surgery. Resume >24-72 h postoperatively, depending on bleeding risk.	Hold ≥24 h before neuraxial or deep nerve blocks.	Hold ≥24 h before neuraxial block; restart ≥4 h after catheter removal. Indwelling catheters are contraindicated with twice-daily low-dose and high-dose LMWH.
Unfractionated Heparin (Subcutaneous)	Not addressed	Interruption not needed routinely; Follow ASRA/ESRA guidelines if neuraxial anesthesia is planned	Not addressed	Not addressed	Low dose £200 IU/kg/day SC-Hold 4 h High dose- Hold 12 h before neuraxial or deep nerve blocks.	Hold 4-6 h for low dose or 12-24 h for high dose before neuraxial or deep nerve blocks. Restart immediately following catheter removal.
Unfractionated Heparin (Intravenous)	Not addressed.	Stop 4–6 h before surgery; check aPTT.	Not specifically detailed.	Stop 4–6 h before procedure; resume 24 hours after procedure	4–6 h before neuraxial or deep nerve blocks.	Hold 4–6 h and ensure normal aPTT before neuraxial/deep nerve blocks; restart ≥1 h after catheter removal.

In many care pathways, these decisions are made before an anesthesiology evaluation, and interruption strategies calibrated solely to surgical bleeding thresholds may inadvertently constrain anesthetic options. Abbreviated interruption intervals that are acceptable for operative hemostasis may leave sufficient residual anticoagulant activity to render neuraxial or deep regional instrumentation inadvisable [8-10]. For example, in a patient receiving a DOAC with severe obstructive lung disease undergoing lower limb surgery with low bleeding risk, a PAUSE or ACCP-recommended interruption of one to two days may effectively exclude neuraxial anesthesia as a viable option. In such circumstances, anesthetic feasibility is effectively narrowed before formal anesthesia assessment has occurred. For selected high-risk populations such as frail older adults undergoing hip fracture surgery, neuraxial techniques may influence outcomes such as pulmonary morbidity, opioid exposure, and recovery trajectories, where anesthetic technique may not merely be a choice but may be outcome-relevant [11]. The converse also merits consideration. Prolonging interruption solely to preserve neuraxial feasibility may extend time off anticoagulation, although the consequences of such prolongation of interruption are currently unknown. Interruption decisions may thus benefit from a multidisciplinary approach so that perioperative anticoagulation management and anesthetic planning are not competing but are, in fact, evolving together.

**Figure 1:**
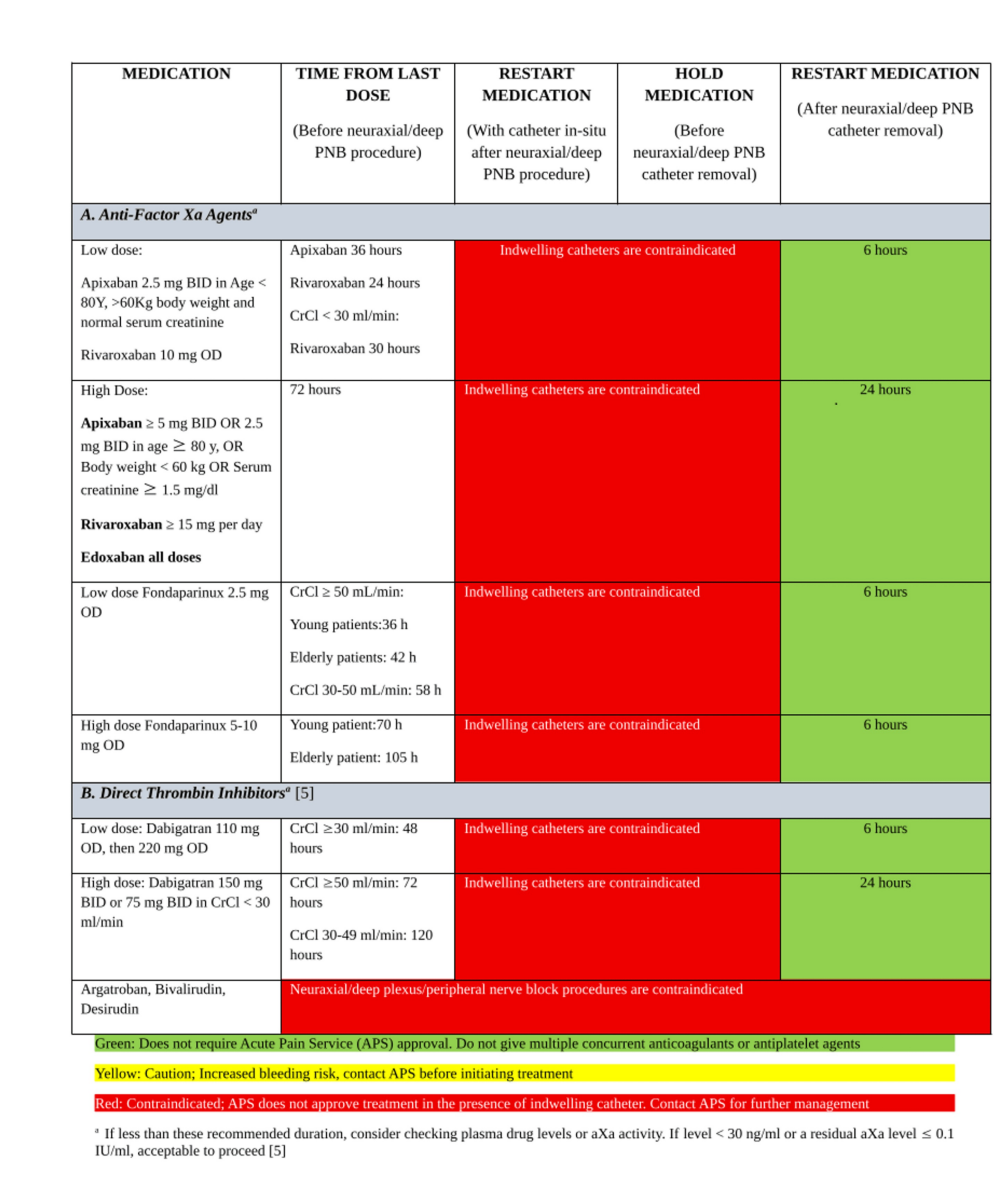
Summary of pause times of anti-Xa and direct thrombin inhibitors based on ASRA guidelines when planning regional anesthesia PNB: Peripheral Nerve Block; BID: Twice a Day; Y: Years; OD: Once a Day; CrCl: Creatinine Clearance; min: Minutes; h: Hours; ASRA: American Society of Regional Anesthesia and Pain Medicine; APS: Acute Pain Service Adapted from [5].

**Figure 2:**
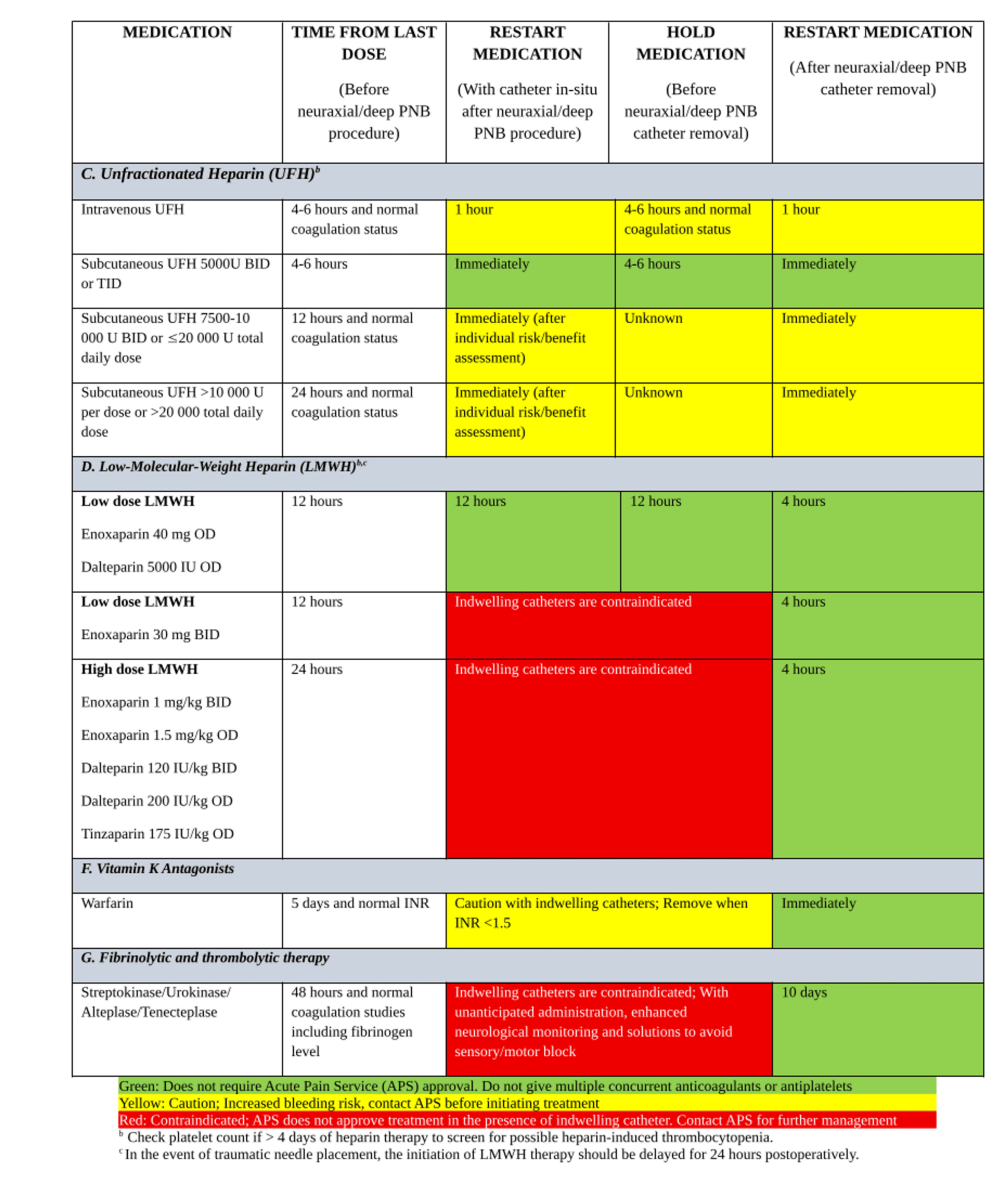
Summary of pause times for heparins, vitamin K antagonists, and fibrinolytics based on ASRA guidelines when planning regional anesthesia PNB: Peripheral Nerve Block; BID: Twice a Day; Y: Years; OD: Once a Day; INR: International Normalized Ratio; min: Minutes; h: Hours; ASRA: American Society of Regional Anesthesia and Pain Medicine; APS: Acute Pain Service Adapted from [5].

**Figure 3:**
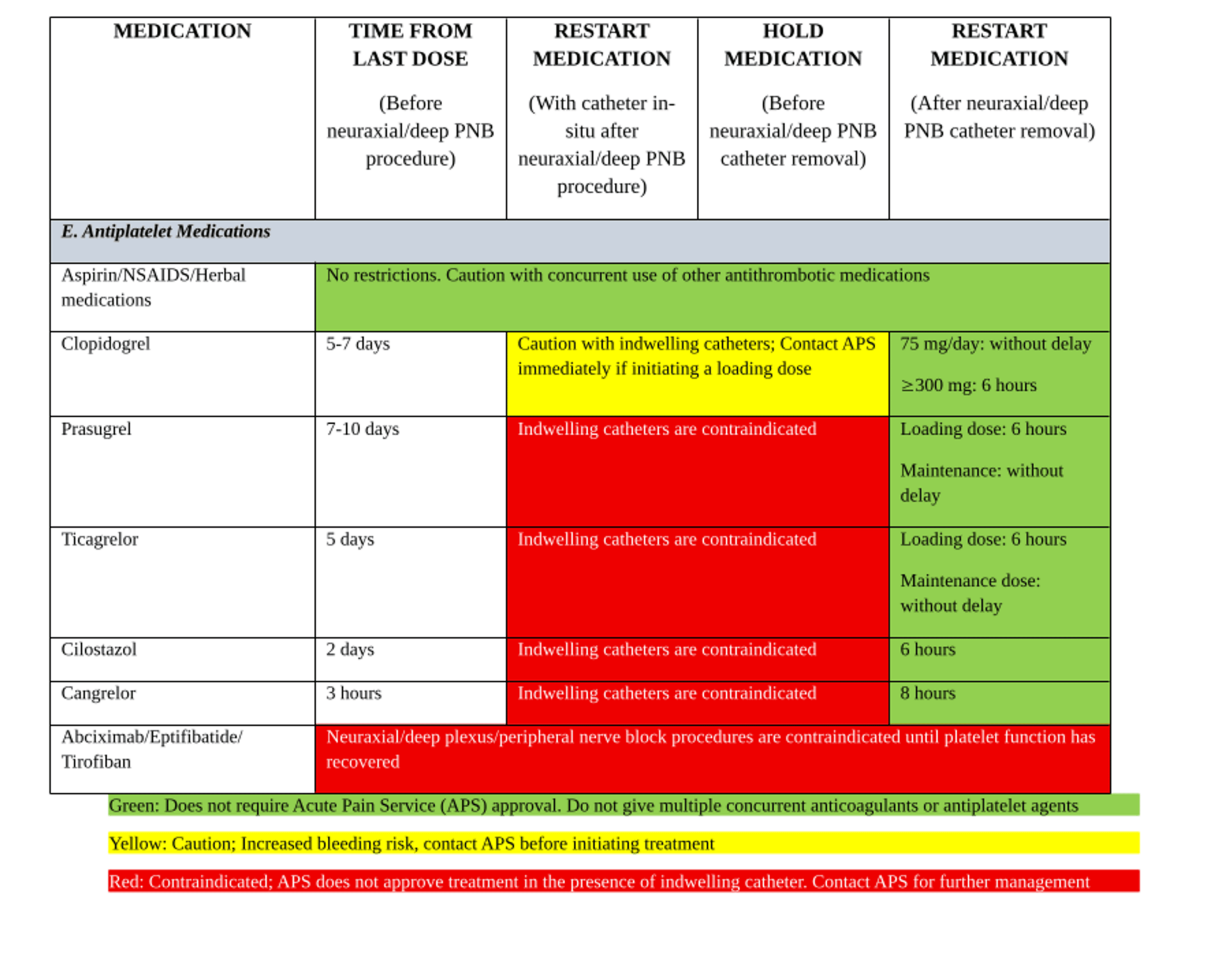
Summary of pause times of antiplatelet medications based on ASRA guidelines when planning Regional Anesthesia PNB: Peripheral Nerve Block; NSAIDs: Nonsteroidal Anti-inflammatory Drugs; APS: Acute Pain Service; LMWH: Low Molecular Weight Heparin; ASRA: American Society of Regional Anesthesia and Pain Medicine Adapted from [5].

A harmonization of multiple existing guidances may be beneficial through institution-specific tools developed collaboratively among anesthesiology, surgery, internal medicine, pharmacy, and preoperative services, and such an example is provided in Figure 1 on the management of anti-factor Xa and direct thrombin inhibitors when considering RA, Figure 2 on RA considerations in the context of heparins (unfractionated and low molecular weight heparin), fibrinolytics, and vitamin K antagonists; and Figure 3 on the considerations for RA in the context of antiplatelet medications.

The intention is not to make this the primary guidance for anticoagulation pauses, but to allow surgical and perioperative specialties to provide guidance on RA feasibility when RA is an anesthetic possibility. Such templates, while institution-specific, may better standardize care in the context of multiple and at times conflicting guidance on anticoagulation pauses. Such institutional policies may reduce scheduling errors and case delays and may even provide guidance on selective physiologic assessment in defined outlier scenarios such as advanced age, renal dysfunction, uncertain last dose timing, or shorter-than-recommended interruption. Time-based algorithms should remain foundational; however, confirmatory laboratory evaluation of residual anticoagulant activity in carefully selected cases may assist teams in aligning surgical bleeding tolerance with neuraxial safety.

Standardized pause intervals remain indispensable. Like fasting guidelines, they provide clarity and reproducibility for most patients. However, fixed intervals do not eliminate physiologic variability. Renal impairment, frailty, polypharmacy, uncertain medication timing, and urgent surgery introduce heterogeneity not fully addressed by time alone. Observational data suggest that a minority of patients retain measurable DOAC levels despite guideline-consistent interruption, particularly among older or higher-risk cohorts [8-10]. While time-based algorithms remain foundational, emerging evidence will inform whether we are moving towards confirmatory laboratory evaluation of residual anticoagulant activity, as with older anticoagulants, for either surgical bleeding tolerance or the safety of RA procedures. In an era of multidisciplinary perioperative medicine, harmonization must be collaborative, not hierarchical. Anesthetic safety should be an integral component of perioperative anticoagulation management.
